# Supplementary material for: Long-term effects of neonicotinoid insecticides on ants
Source: Commun Biol. 2020 Jun 26;3:335. doi: 10.1038/s42003-020-1066-2 (PMC7320190; doi:10.1038/s42003-020-1066-2)
Supplement: Supplementary file 1 — Supplementary Information [file 42003_2020_1066_MOESM1_ESM.pdf]

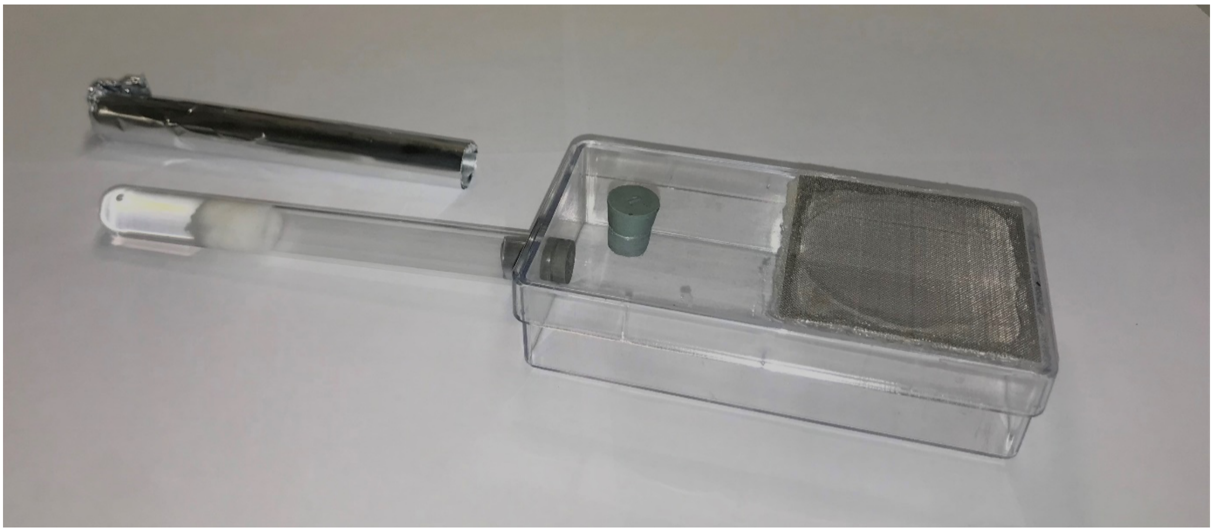

Supplementary Figure 1: Experimental cages. Nesting tube (155 mm length, 14 inner diameters) attached to a foraging arena (135x68x32 mm). The tubes were separated into two compartments with a cotton wool ball. The front chamber housed the colonies and the rear chamber was filled with water (controls) or thiamethoxam treatment solutions (low = 4.5 µg/L, high = 30 µg/L).

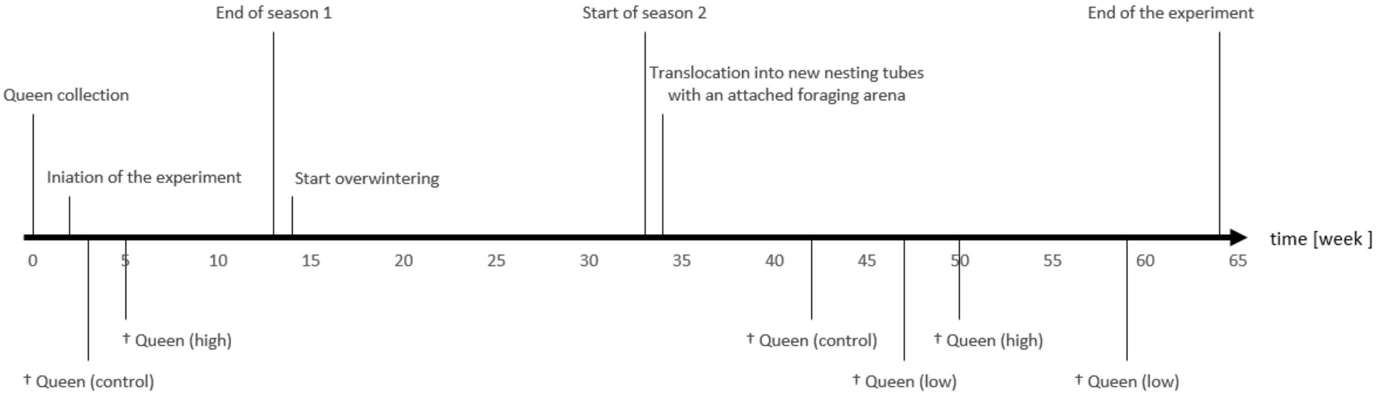

Supplementary Figure 2: Experimental timeline. Timeline of events starting with the collection of queens (Week 0) until termination (week 64), including the death of queens (†). Upon initiation of the experiment, colonies were exposed chronically to thiamethoxam (controls = 0 µg/L, low = 4.5 µg/L, high = 30 µg/L).
