# Supplementary material for: Long-term effects of neonicotinoid insecticides on ants
Source: Commun Biol. 2020 Jun 26;3:335. doi: 10.1038/s42003-020-1066-2 (PMC7320190; doi:10.1038/s42003-020-1066-2)
Supplement: Supplementary file 2 — Description of Additional Supplementary Files [file 42003_2020_1066_MOESM2_ESM.pdf]

Description of Additional Supplementary Files

File Name: Supplementary Data 1
Description: Data supporting the findings of the study “Long-term effects of neonicotinoid insecticides on ants”
