# Supplementary material for: Long-term effects of neonicotinoid insecticides on ants
Source: Commun Biol. 2020 Jun 26;3:335. doi: 10.1038/s42003-020-1066-2 (PMC7320190; doi:10.1038/s42003-020-1066-2)
Supplement: Supplementary file 4 — Reporting Summary [file 42003_2020_1066_MOESM4_ESM.pdf]

nature research

Corresponding author(s): Daniel Schläppi

Last updated by author(s): May 18, 2020

Reporting Summary

Nature Research wishes to improve the reproducibility of the work that we publish. This form provides structure for consistency and transparency in reporting. For further information on Nature Research policies, see Authors & Referees and the Editorial Policy Checklist .

Statistics

For all statistical analyses, confirm that the following items are present in the figure legend, table legend, main text, or Methods section.

n/a	Confirmed	
☐	☒	The exact sample size (*n*) for each experimental group/condition, given as a discrete number and unit of measurement
☐	☒	A statement on whether measurements were taken from distinct samples or whether the same sample was measured repeatedly
☐	☒	The statistical test(s) used AND whether they are one- or two-sided<br>*Only common tests should be described solely by name; describe more complex techniques in the Methods section.*
☒	☐	A description of all covariates tested
☐	☒	A description of any assumptions or corrections, such as tests of normality and adjustment for multiple comparisons
☐	☒	A full description of the statistical parameters including central tendency (e.g. means) or other basic estimates (e.g. regression coefficient) AND variation (e.g. standard deviation) or associated estimates of uncertainty (e.g. confidence intervals)
☐	☒	For null hypothesis testing, the test statistic (e.g. *F*, *t*, *r*) with confidence intervals, effect sizes, degrees of freedom and *P* value noted<br>*Give P values as exact values whenever suitable.*
☒	☐	For Bayesian analysis, information on the choice of priors and Markov chain Monte Carlo settings
☒	☐	For hierarchical and complex designs, identification of the appropriate level for tests and full reporting of outcomes
☒	☐	Estimates of effect sizes (e.g. Cohen's *d*, Pearson's *r*), indicating how they were calculated

Our web collection on statistics for biologists contains articles on many of the points above.

Software and code

Policy information about availability of computer code

Data collection	No computer code or software was used to generate data. The data is based on observations of laboratory Lasius niger colonies.
Data analysis	Only publicly available software (Custom code in R + associated packages; cited and referenced in the manuscript) were used for the data analyses (Standard statistical approaches described in material and methods).

For manuscripts utilizing custom algorithms or software that are central to the research but not yet described in published literature, software must be made available to editors/reviewers. We strongly encourage code deposition in a community repository (e.g. GitHub). See the Nature Research guidelines for submitting code & software for further information.

Data

Policy information about availability of data

All manuscripts must include a data availability statement. This statement should provide the following information, where applicable:

- Accession codes, unique identifiers, or web links for publicly available datasets
- A list of figures that have associated raw data
- A description of any restrictions on data availability

Data supporting the findings of this study are available fin the supplementary materials.

Field-specific reporting

Please select the one below that is the best fit for your research. If you are not sure, read the appropriate sections before making your selection.

☐ Life sciences ☐ Behavioural & social sciences ☒ Ecological, evolutionary & environmental sciences

Ecological, evolutionary & environmental sciences study design

All studies must disclose on these points even when the disclosure is negative.

Study description	Schläppi et al. report on a laboratory experiment, where they look at long term effects of chronic thiamethoxam (neonicotinoid insecticide) exposure on the colony development of black garden ants (Lasius niger)
Research sample	Most studies on sublethal effects have focused on pollinators due to their economic importance. However, little research exists on long-term consequences of chronical insecticide exposure to long-lived soil-dwelling organisms. Regarding long life spans, ant-queens are outstanding, especially black garden ant queens that can live up to 30 years. L. niger is endemic to Europe and colonies can be regarded as sedentary, potentially leading to exposure over decades. Thus, L. niger was chosen as study species.
Sampling strategy	Gynes of L. niger were captured after their nuptial flight and then exposed to field-realistic sublethal levels of thiamethoxam for for two developmental seasons until the second overwintering. Due to logistical reasons involved in keeping colonies for such a long duration, 30 queens were assigned to three thiamethoxam treatments (control = 0 µg/L, low = 4.5 µg/L, high = 30 µg/L; N=10 each).
Data collection	Data collection is described in the material and methods section: D.S. and N.K. conducted performed the experiments and collected the data. Colony monitoring was performed by N.K. during the first season. Counts and measurements before the second overwintering as well as the UHPLC-MS/MS analyses were done by D.S..
Timing and spatial scale	Timeline of the most important events can be found in supplementary figure 1. Start is the collection of queens after their nuptial flight (30.07.2016, Week 0) . Colony development was then monitored until the second overwintering (Week 64).
Data exclusions	No data were excluded.
Reproducibility	The experimental set up is described in the material and methods section and could be reproduced by other research groups. Data supporting the findings of this study are available from the corresponding author in order to reproduce the results from the collected raw data.
Randomization	Colonies were randomly assigned to the three treatments based on a random number generator created as custom code in R.
Blinding	N/A

Did the study involve field work? ☒ Yes ☐ No

Field work, collection and transport

Field conditions	The fieldwork consisted of sampling queens after mating flight. Nuptial flights always take place at the same time of year (late June-early July) and the same time of day (late afternoon), and occur during periods of hot weather just before or following rainfall.
Location	Bern , Switzerland
Access and import/export	The ants were collected on a concrete place just outside a supermarket (accessible to general public).
Disturbance	Apart from the collection stress induced on captured queens no further disturbance was caused.

Reporting for specific materials, systems and methods

We require information from authors about some types of materials, experimental systems and methods used in many studies. Here, indicate whether each material, system or method listed is relevant to your study. If you are not sure if a list item applies to your research, read the appropriate section before selecting a response.

Materials & experimental systems

n/a	Involved in the study	
☒	☐	Antibodies
☒	☐	Eukaryotic cell lines
☒	☐	Palaeontology
☐	☒	Animals and other organisms
☒	☐	Human research participants
☒	☐	Clinical data

Methods

n/a	Involved in the study	
☒	☐	ChIP-seq
☒	☐	Flow cytometry
☒	☐	MRI-based neuroimaging

Animals and other organisms

Policy information about studies involving animals; ARRIVE guidelines recommended for reporting animal research

Laboratory animals	Drosophila hydeii used for feeding the ant colonies were purchased from Qualipet (Vet-store, Switzerland).
Wild animals	The study did involve field collected Lasius niger queens that were used to raise laboratory colonies.
Field-collected samples	Lasius niger queens were collected after their nuptial flight. Colonies were then raised in nesting tubes (Supplementary Figure 1; 155 mm length, 14 mm inner diameter) with a cotton wool ball separating it into two compartments. The rear chamber was filled with one of the three treatment solutions (10 ml). The first cell housed the colony and was closed by another cotton wool plug. Nesting tubes were wrapped in aluminium foil, maintained at RT (19-23°C) and protected from sunlight. Week 34 the colonies were transferred into new nesting tubes with freshly prepared treatment solutions and an attached foraging arena (135x68x32 mm). Once the first workers emerged in the first season, colonies were provided twice with 30 µl droplets of 1:1 sugar-water. In the second season, the ants were provided weekly with a 1:1 sugar-water drenched cotton ball and drosophila flies (Drosophila hidey). In July 2016, we switched from Drosophila to honeybee (Apis mellifera) pupae as a protein source to satisfy the increased protein needs of the colony.<br><br>Apis mellifera pupae used for feeding the colonies were freshly sampled from local honeybee colonies of the institute apiary (Institute of Bee Healh, Bern Switzerland) and frozen at -80°C until use.
Ethics oversight	No ethical approval was required to work with these invertebrate species

Note that full information on the approval of the study protocol must also be provided in the manuscript.
